# Developing a comprehensive school-based physical activity program with flexible design – from pilot to national program

**DOI:** 10.1186/s12889-020-10111-x

**Published:** 2021-01-07

**Authors:** Kerli Mooses, Triin Vihalemm, Marko Uibu, Katrin Mägi, Leene Korp, Maarja Kalma, Evelin Mäestu, Merike Kull

**Affiliations:** 1 Institute of Sport Sciences and Physiotherapy, Faculty of Medicine, University of Tartu, Ujula 4, 51008 Tartu, Estonia; 2 Institute of Computer Science, Faculty of Science and Technology, University of Tartu, Narva mnt 18, 51009 Tartu, Estonia; 3 Institute of Social Studies, Faculty of Social Sciences, University of Tartu, Lossi 36, 51003 Tartu, Estonia

**Keywords:** Physical activity intervention, School, Children, Intervention design

## Abstract

This article focuses on the process of designing the vital, participatory school-based intervention program aiming to increase the physical activity in schools. The program analyzed is Estonian nationwide comprehensive physical activity program Schools in Motion (SiM) that recently received European Commission’s #BeActive Education Award. The program has a good performance in terms of willingness of schools to participate in co-creation of program development, the high interest to join the program and zero dropouts, and strong partnership with ministries which enables to actively participate in policy making. Authors analyze the key elements of the planning, piloting, implementation, and scaling phases of the SiM program and share their lessons learnt in co-working with schools. The difficulties faced during the development process, the strengths and challenges associated with an interdisciplinary approach, and involvement of schools as experts have been addressed.

## Background

Physical activity (PA) is an important lifestyle factor associated with a wide range of benefits in children’s health and development, including the prevention of overweight, obesity, and cardiovascular diseases as well as supporting academic achievement and mental health [1–3]. It has been pointed out that children who are not participating regularly in structured motor-skill-enriched activities may never reach their genetic potential for motor control that underlies sustainable physical fitness later in life [4]. The World Health Organization (WHO) recommends children to be engaged in moderate to vigorous PA (MVPA) at least 60 min per day [5]. However, studies with objectively measured PA suggest that only 4.6% of girls and 16.8% of boys in Europe aged 10–12 years meet the current PA recommendations [6], and these inactivity trends also dominate in Estonia [7].

Kohl et al. [8] have stated that “the pandemic of physical inactivity should be a public health priority”. At the same time, it has been stressed that multilevel and multisector plans are needed and all sectors outside the health sector must be involved in the fight against physical inactivity [9, 10]. Reis and colleagues [10], after studying numerous PA interventions, have called for action-oriented research addressing the scalability of interventions that can work in real-world settings. They note critically that PA interventions, even when proven to be effective, remain short-lived because they fail to become embedded in a system once the research funds have expired [10]. Our experience offers information how to make the PA intervention programs more viable [11] and increase their provisional stability [12] - that is to prolong the impact of the intervention both in terms of its utility for target groups and its resilience: the continuation of the existence of desired social practices and their embeddedness to the social fabric at the site of intervention.

Schools are potentially powerful agents for changes to support PA levels [13] but there is a need for a flexible approach and empowerment, as PA promotion is not an inherent part of their existing agenda as PA in school is sparse and is often restricted with physical education lesson [14]. Therefore, it is a challenge to involve and empower schools to become efficient agents of PA enhancement and to ensure that the PA-related practices are embedded into the system. Similar to other institutions undertaking PA interventions [10], the schools face a scarcity of intervention design descriptions that: (a) would combine practice-to-evidence with evidence-to-practice; (b) would be scalable without substantial financial support; and (c) address the complexity of planning and implementation process. While there is documentation concerning the development and content of several PA interventions which are essential to enable other researchers to understand why interventions do or do not work, there is only limited data available about the development process of comprehensive school-based PA intervention programs [15]. Current article addresses the issues related with participatory, action-research oriented intervention program that was designed together with schools and what shows high motivation to join and no dropouts among schools. Authors - research and development team (RDT) of the University of Tartu - reflect the design process of comprehensive PA intervention program for Estonian schools – Schools in Motion (SiM): its planning, piloting, implementation, and scaling phases. Their critical self-reflection is condensed into the section of “Lessons learnt and primary indicators of success” that were put together collectively by the RDT members. The overview is based on constant action research, the discussions and revision of different data sources such as documents, progress reports/papers, individual diaries, and observation notes. To recognize critical elements in the design process program, several extensive brainstorming sessions and discussions were held. Collaborative writing by all the authors was applied where the perspectives and major insights were discussed, written, and re-written. We relied also on the documents and individual notes we have taken during the process which helps to lessen the distortive effect of retrospective analysis and hindsight biases. Although inherently and inevitably subjective to a certain extent and not able to convey the full complexity of the design process, the methodological approach we followed in this study gives a broad basis for analytical conclusions and suggestions. The empirical evidence collected during the action research and development process has not been published yet. The measurement of social impact of the action-research based PA program where the actions change continuously based on the feedback from participants, needs further attention and deserve separate article.

Current article is focusing on the design of the socially viable school-based PA intervention programs with the aim to inspire and offer tips for designing and implementing PA-enhancing interventions within school systems.

## Main text

### The framing of the program

The SiM program has been successfully scaled up, starting from 10 pilot schools in 2016 and reaching 110 schools in 2020. The SiM program is currently targeted at basic school (grades 1–9, ages 7–16 years). The group of schools who participate in the program is diverse in terms of size and location, involving rural schools with 15 students up to urban schools with more than 1300 students. The Estonian SiM program has received a great deal of attention in both Estonia and abroad. For example, SiM won the European Commission’s #BeActive Education Award in 2019, which recognizes activities in the field of education to encourage young people to be more physically active [16].

**Fig. 1:**
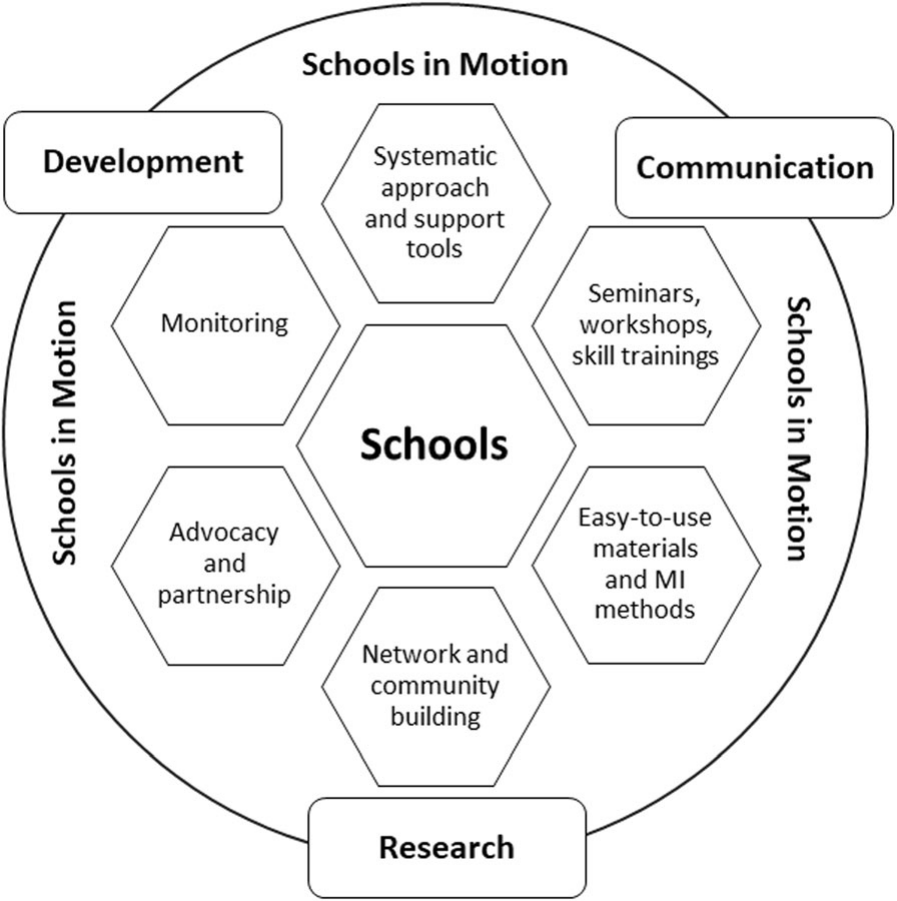
Schools in Motion (SiM) program activities from the research and development team’s viewpoint

The Estonian SiM program aims to achieve sustainable change towards a PA-friendly mode of everyday operation in schools, similarly to “Whole School” approaches to PA-intervention that have arisen from Toronto Charter [17], and Comprehensive school physical activity program (CSPAP) [18]. We conceptualize the intervention as principal social change in a pro-sedentary school system. We consider that our task is not limited to creating changes to a few specific activities but involves multi-faceted action-research of possibilities to transform current meanings and understandings, skills and knowledge, things and infrastructure so that the practices are embodied during the school day [11]. Accordingly, SiM has been developed as a comprehensive and flexible PA program which supports the participating schools in redefining and designing the schools’ practices and conditions in a PA-supportive way and in offering more PA opportunities for students and personnel through a systematic approach. Participating schools are supported by seminars, workshops and skills training for school personnel and students. Moreover, easy-to-use materials, advocacy in changing the social norms, a supportive network and action research are provided by the research and development team (RDT) through an iterative process with schools and program partners (Fig. 1). However, no material incentive is distributed directly to the schools.

**Fig. 2:**
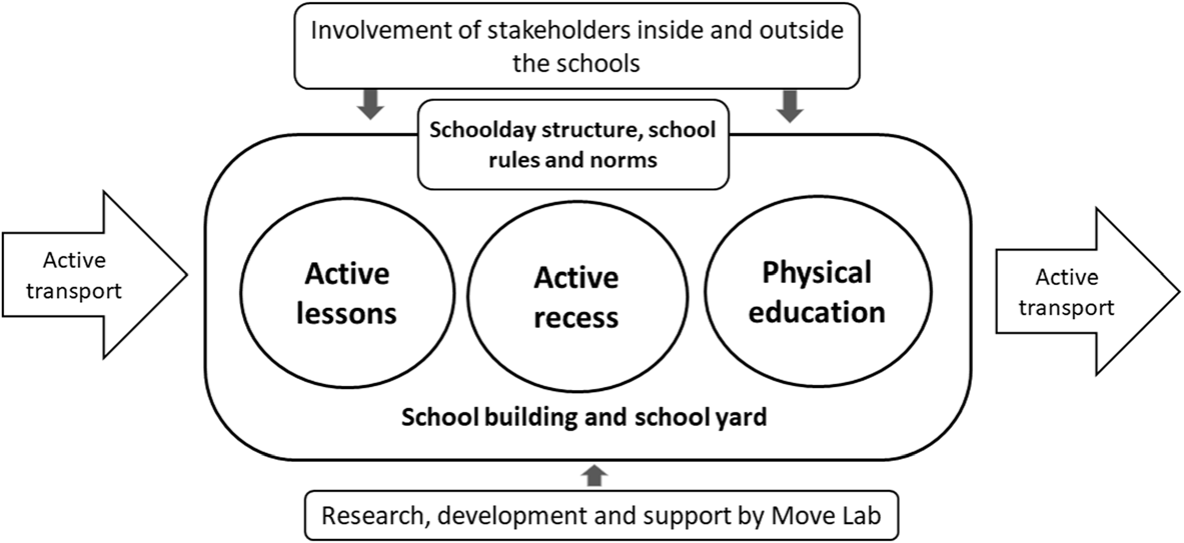
Schools in Motion’s general model from the schools’ viewpoint

Figure 2 depicts the schools’ perspective of SiM: the intervention field with inputs from RDT and stakeholders. The model is based on a comprehensive approach, outlining the temporal “venue” for the intervention: the school day as a whole, beginning with transport to school, and continuing with framing the structure of school day with its timetables. The model emphasizes the involvement of school personnel, students and parents; providing opportunities for PA and reducing sedentary time during academic lessons and recess; renewing the physical education; supporting changes in the indoor and outdoor environment, and the development of new methods and monitoring of the changes by the RDT (presented more concisely in Fig. 1).

Moreover, the renewing of the physical education curriculum, where the sport-centered approach interchanged with the lifestyle-centered approach, is included. The renewing of physical education is an ongoing process but is not discussed in the current analysis. Finally, the schema highlights the role of different stakeholders and interest groups who should participate in the process to achieve appropriate, meaningful and sustainable results.

During the implementation of the program, this model has maintained both a structuring and presentation function, although the elements depicted in the model are not presented equally in the program. Throughout the process it has been important to support the autonomy of the schools, e.g. every school creates their own action plan and schedule, schools are free to decide their aims and actions while the SiM model and program activities provide them with certain tools and general suggestions. All schools entering into SiM program participate in one-day program training “Start-up seminar” with school team (max 5 members, including the school principal), after that several training seminars and workshops are made available for teams, teachers, and students. Schools are free to choose seminar or workshop to participate in depending on their own action plan, needs and main focuses. To support the autonomy of the schools and prevent overload associated with project activities, there is no mandatory seminar schedule for schools. Generally, most schools have been highly motivated to participate in provided trainings and seminars.

### Development and design process of SiM

**Fig. 3:**
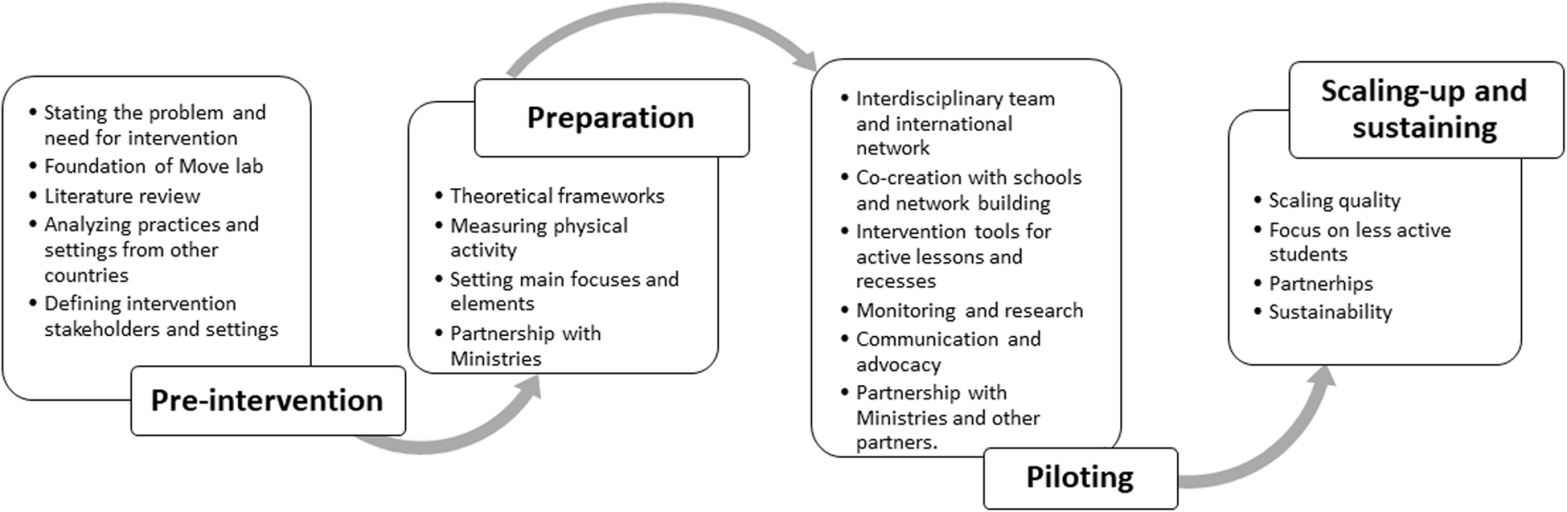
The phases and main focus activities in development process of the Schools in Motion (SiM) program in 2014–2020

The development process in 2014–2019 can be divided into four main phases which main focuses and activities are presented in Fig. 3.

#### Pre-intervention

##### Stating the problem and the foundation of the move lab

From 2014, we started to focus on the problem of children’s physical inactivity as a serious risk for public health and wellbeing given that, according to a national survey, only 16% of 11- to 15-year-old students met the PA recommendations every day in Estonia [19]. We stated that there is a serious need for action and we applied for funding to begin a systematic process towards intervention. The financial support received from the Research Innovation Foundation of University of Tartu enabled us to form the specialized unit Move lab in the Institute of Sport Sciences and Physiotherapy with the purpose of developing an evidence-based PA intervention program for children.

##### Literature review, analyzing previous practices and defining stakeholders and setting

Based on the scientific literature, we concluded that it is important to start the intervention from the youngest age groups, as the period between 6 and 12 years is critical in forming healthy lifestyle behavior and PA habits [20]. The analysis of the existing scientific literature indicated that one of the most promising settings to influence the PA levels of students is the school [13, 21], thus the school was selected as an intervention venue. Schools are prospective sites for systematic pro-PA change because they enable us to engage children and families across the social spectrum and reveal a strong potential as central institutions in a community that provides participation opportunities for a critical amount of PA [13]. The next step was to map different components that previous school-based PA interventions had used, and which have a potential effect of the PA levels of students [13, 20]. We concluded that it is important to focus on the development of a multicomponent approach as one component of the intervention (e.g. interventions only during lessons or in recess) might not be sustainable as they are not usually accompanied by changes in school culture [18, 22] and the lack of support by school administrations can be one hindering factor in the implementation of the intervention [22].

#### Preparation phase

##### Deliberation of theoretical frameworks

Special focus was given to theoretical frameworks that could have been used in the development process. A socio-ecological model [23] and components of self-determination theory [24] were taken as general supportive theoretical frameworks. Self-determination theory has been used in previous interventions to explain the role of social factors (e.g., autonomy-supportive and controlling behavior) on individuals’ motivation via three basic psychological needs for autonomy, competence and relatedness [24]. The socio-ecological model [23] not only helps to target factors on an individual level, but also an interpersonal and organizational level. The planning of the development process was started based on an Intervention Mapping (IM) approach, which consists of a six-step protocol that facilitates a stepwise process for theory- and evidence-based development of health promotion interventions [25]. During this process we met the same difficulties that have been pointed out by other researchers – IM’s limitation is its time-consuming nature [26], and, as IM is typically applied to simple and uni-dimensional behaviors, IM is unfeasible and impractical when applied to multi-dimensional behaviors [27]. At the beginning of the process the main focus was on the PA behavior of the children and the empowerment of teachers. After many discussions and brainstorming sessions, we understood there is a need to broaden our focus and target additionally changes in school culture as well as general social norms. Therefore, the program framework was redesigned based on the practical guide of planning social change programs [11] developed on the grounds of theoretical conceptualization of a change of social practices [28]. In this conceptualization, the focus of inquiry and intervention shifts to social practices that are re-enacted and co-constituted by the actors and the socio-material structures [28, 29]. The conceptualization de-centres the individual [30] and integrates the context [29], thus, partly withdrawing from the socioecological model. With these choices, the SiM program distanced itself from “Whole School” approaches and CSPAP. In addition, the aspect of vitality, i.e. how to embed it into the (school) system, which is crucial for sustainability [10], was emphasized.

##### Measuring physical activity

As there was a lack of objectively measured PA data in school settings, we carried out a nationwide objective PA measurement among 7- to 13-year-old students, where 819 students from 13 schools wore the accelerometer for seven consecutive days [7]. According to the results, although 24% of students met the PA recommendation on school days, there were 17% of students who did not meet the PA recommendation on any of the school days and 18% who met the PA recommendation on only one school day [7], indicating a significant proportion of inactive students who are at health risk. The study also revealed that the academic lessons were very inactive and uninterrupted sedentary time was dominant [31], while in physical education lessons only 13 min of MVPA was acquired [32]. As a result, only 3% of students acquired at least 30 min of MVPA (which is half the daily recommendation) during school hours [7]. These findings strengthened our understanding that the school setting needs to be targeted. In addition, small-scale pilot studies to test the applicability of active recess [33] and active lessons [34] were carried out. These studies confirmed that activity breaks in lessons and active recess are promising elements in the whole program to create PA opportunities in the school.

##### Setting main focuses and elements of SiM

Additionally, we carried out focus group interviews in three Estonian schools with varied sizes, locations, and opportunities for PA. We conducted 17 focus group interviews with 92 children aged 8–15 and 9 focus group interviews with teachers, school principals and parents. The aim of our qualitative research was to understand the perceptions of different stakeholders about PA in school settings and to identify how they evaluate the possibilities for PA, what the existing practices are, and whether they feel a necessity for change considering PA opportunities in school [35, 36]. Interviews with different participants indicated that the meaning of PA was strongly based on a sport paradigm, which was considerably limiting options for moving, and was alienating some students who did not have a good relationship with sports. When interviewees talked about PA, they immediately brought in the distinction of ‘good’ and ‘bad’ students in PA. For example, sport-friendly families spend all their time hiking, running and cycling, participating in sport events, training sessions, etc., while there are those lagging behind – inactive children who are not willing to walk to school even if it takes 10 min, so-called ‘couch-potatoes’ [36]. Considering the school day, the students found that their PA is limited during recess, and accordingly a non-supportive physical and organizational school environment, e.g. lack of appropriate areas, facilities, and equipment, a lack of time as the recess is short (usually 10 min), and restrictive regulations (e.g. not allowed to run indoors) [35]. Describing academic lessons, the activity breaks in lessons or integrated PA with the lesson context were not common and were practiced by only a few specific teachers [36]. There was a strong willingness and desire to be more active in lessons by the students, while the main barriers for teachers to involve PA in their lessons were the lack of skills, tools and/or motivation.

The interviews indicated that overall attitude for PA is positive in schools. However, there is a need to initiate a change in school culture through: 1) creating new practices, e.g. possibilities for PA during recess and lessons by introducing new activities; 2) changing the meaning of PA, e.g. movement during the school day is normal and PA can support mental effort and academic achievement, PA is not only sport; 3) developing the skills of teachers and students, e.g. applicable techniques and methods to integrate PA to academic lesson and recess; and 4) providing supportive tools, e.g. materials/ideas for changing the physical environment [36]. According to these needs, a general model for SiM was elaborated (presented in Figs. 1 and 2).

##### Establishing a partnership with ministries

In parallel with the creation of the program model, negotiation with the Ministry of Social Affairs and the Ministry of Education and Science started to inform them about the serious situation concerning the low levels of PA in children and the framework of evidence-based solutions was provided. Positive feedback from the ministries for initiating piloting was received. For more wide partnership a nationwide forum “Children’s physical activity levels are decreasing – how can we stop that?” was organized with the purpose of calling society, different activity groups and politicians to action.

#### Piloting phase

In 2016, the piloting of the SiM program received encouragement from the Ministry of Social Affairs and funding from the Council of Gambling Tax. The RDT sent out the call to participate in the SiM pilot program. In order to participate, schools had to send a motivation letter. Based on 18 received motivation letters, 10 schools of different size (per student), location (urban, rural), existence of outdoor area (yes/no) and number of students (min 87, max 936 students) were selected.

Although the plan was to run the pilot phase with 10 schools from 2016 to 2019, more schools joined in during this period due to the strong interest from schools and additional funding, culminating in 78 network schools by 2019.

##### Forming the interdisciplinary team and international network

As the problem targeted by SiM is multifaceted and complex, an interdisciplinary team was formed from experts from sport and health sciences, education, social sciences, psychology and communication. The leading institution was the Institute of Sport Sciences and Physiotherapy, with experts who had previous experience with applied projects. Two non-academic consultants were invited as consulting members of the team as they had previously led a smaller-scale project called Safe and Active School Day. Their existing contacts as well as expertise in educational settings proved highly valuable in, for example, deciding how to communicate with or to motivate schools (see Tip 1 in Table 2).

During the process it became clear that the instrumental distribution of specific roles/tasks was not functional as there were conflicting views regarding some fundamental principles about the logic of intervention. In the case of transdisciplinary projects with multiple participants, it is not surprising that setting priorities and agreeing upon the best approaches are complicated processes. Inevitably, the experts of transdisciplinary projects have their own priorities, understandings, incommensurable experiences, background knowledge and practical considerations that might be conflicting or at least complicate the goal of reaching a common understanding or agreement [37, 38]. For SiM, it was the empirical evidence and close contact with schools that helped to clarify the framework of the pilot program. Discussions regarding the basic principles of the responsibilities of individuals, and about the social marketing and co-design approaches reached, led to the acknowledgement regarding the autonomy of schools.

Members of the SiM program team had significant differences in the paradigmatic approach and program design process. Therefore, it was challenging for the RDT to mix the different disciplines. Additionally, first meetings with school members indicated that the real life is much more nuanced than the academic disciplines prescribe. The diverse interpretations from numerous theoretical paradigms and practical angles were considered, all without giving priority to the academically “elegant” explanations – this is also called democratization of academic paradigmatic knowledge [39]. Thus, both the research based on the individuals’ motivation via three basic psychological needs for autonomy, competence and relatedness [24] and practice-theory-based research (described above) can be found in mutually productive dialogue. In general, albeit through the course of several debates and contradictions in views, the team has learned that a transdisciplinary approach is a great strength as it will not allow us to make simplifications and assumptions based on a limited disciplinary view. The common aims to strive for are largely pragmatic – to empower the schools with knowledge-sharing in varied forms, such as mutual visits, seminars, training sessions, and inspiration days, as described in the following sections.

Additionally, the need for international collaboration was stated, and we contacted the LIKES Research Centre from University of Jyväskylä in Finland. LIKES had developed the nationwide PA program “Liikkuva Koulu” (Finnish schools on the move), which has to a large extent succeeded in Finland. Their program’s approach differs from many others as it is a so-called “bottom-up” approach, which supports schools’ own autonomy [40]. We can conclude that the cooperation in sharing research and practical issues has been very fruitful, supportive and necessary to solve problems and build up interventions that are not just regional but global. International scientific cooperation has widened throughout the pilot period with other Nordic and Baltic countries, and this is still an ongoing process.

##### Co-creation with schools and network building: school visits, supporting schools’ team building, schools’ action plans and schools’ networking

During the planning phase of the SiM program, there was intensive debate regarding whether or not to include school visits to the program as they were considered too time-consuming and intrusive. However, a decision was made to test it and, during the first months of the pilot program, all schools were visited by the RDT. This was an informal visit which enabled us to gain a greater understanding of the schools and their peculiarities. In terms of the program development, it turned out that such school visits were invaluable as they helped us to understand that each school is unique and that it is impossible to develop a one-size-fits-all model. It became more evident that we can support the schools by developing materials and tools, carrying out training, and supporting the exchange of good practices and ideas for different program elements but the schools must have autonomy to choose their own focus and set of tools and methods (see Tips 6, 9 and 10 in Table 2). This approach has also been confirmed by the Finnish program [40].

The central principle of the SiM – the schools are autonomous implementers of the program – means that schools needed broad-based and strong teams. To emphasize the importance of involving staff and the entire school environment, we suggested the participating schools form a 5-person team who would lead the SiM activities in school. The rationale of creating such a team was to support in-school co-operation in planning and implementing SiM activities, for them to support each other and reduce the risk of burnout, and to involve different stakeholders. During the pilot phase we realized that physical education teachers can be involved in the schools’ SiM team but there is a risk of the activities becoming too sport centered and too many implementation tasks being assigned to the physical education teacher. Therefore, when building the team, we suggested that SiM should not become the sport-related duty for physical education teachers but a whole-school endeavor for a variety of PA opportunities.

The piloting years indicated that it is essential that a representative from school management is included in the school team, as support from management is crucial to implement changes. As we promote structural changes, it would be very difficult to achieve them without the involvement of management. In addition, the active roles of principals and head teachers provide a general supportive climate towards PA in school.

All school teams participated in several seminars where they were informed about the aims and opportunities of the pilot program as well as the commitment it would require. School teams were also advised and encouraged to invite children, parents, and a broad range of personnel in the planning and implementation processes in order to ensure a broader circle of people who can carry the ideas of the SiM program and initiate actions in schools. Consequently, the workload is more distributed and the danger of burnout of active leaders alleviated (see Tip 9 in Table 2).

Schools were asked to create an individual action plan for the next school year on how to implement PA into the school day. As each school is unique, it was important to support schools’ autonomy through a flexible approach and enable each school to select elements of their action plan by themselves. However, as a general principle and suggestion, we encouraged them to concentrate on one or two main elements per year in their action plan (e.g. active recess and/or environment or active lessons). The aim was to deter enthusiastic schools from over planning only to realize during the process that they do not have resources to carry out all the started activities (see Tips 4 and 9 in Table 2).

Networking and exchange of practices (both good practices as well as failures) were encouraged and supported throughout the pilot program through different school visits and seminars for school teams on a regular basis. Each year there were at least two seminars for school teams whose aim was to support networking, obtain feedback about project activities and input into further development needs. Throughout the development and piloting process, a bottom-up approach and top-down approach have been used simultaneously. This means that the schools are considered as partners and experts whose feedback is thoroughly considered. At the same time different solutions and elements are developed and disseminated centrally by the program’s RDT. It can be said that schools in the SiM program are not intervention venues but active creators. The schools are also involved in the dissemination process as, in some cases, the experience and advice from other schools is more valued compared to the advice of university researchers (see Tips 1, 5 and 6 in Table 2).

##### Developing intervention tools for active lessons

During the pilot years, among other training sessions and seminars, schools were provided an opportunity to send 4–5 teachers to the training sessions of active lessons. Training seminars were designed, piloted and conducted by RDT. The aim of these seminars was to: 1) increase the awareness of the positive influence of PA on mental health, cognitive functions and learning; 2) provide new skill and exchange experiences; and 3) provide supportive and ready-to-use materials to the participants. The teacher training for active lessons consisted of two training days in order to help to anchor the skills and ideas learnt during the training and for them to gain confidence in using them. On the first training day the importance of PA on learning and academic achievement was highlighted. Throughout the day the techniques for reducing sedentary time and integrating PA into learning were modelled and the teachers had the possibility to play through various activities and discuss with colleagues where and how they could be used with their students. The teachers also received supportive materials and were requested to keep a diary of their practice for two weeks. After a month of individual practice at school, the teachers received the second training day, where the focus was more on the exchanging of experiences as well as getting new ideas to support the teachers’ motivation to carry on with the activities (See Tip 7 in Table 2). Both training days also included an outdoor learning session, as outdoor learning is not very common in Estonia. During the initial pilot phase lesson observations in schools were also conducted during and after the training period. These visits provided an overview of how movement integration works in real classrooms and is implemented by different teachers and was an important part of the co-creation process with the teachers. Selected activities from both the teachers’ diaries as well as observations were made publicly available on the program webpage, where the ideas and methods can be conveniently browsed and searched for.

During the pilot period, 192 teachers from 39 schools participated in the training days. The feedback survey of active lessons’ training showed that, after the training days, 97% of the teachers felt more confident in integrating movement into lessons and movement was more often integrated into the lessons – the proportion of teachers who reported integrating movement into lessons on a daily basis increased from 30 to 61% [41]. Due to a lack of resources not all teachers could attend our training, thus participating teachers are encouraged to share learned methods with their colleagues (see Tips 7 and 8 in Table 2). However, it is helpful when more than one teacher from the same school can participate in the training, so that they can support each other and plan how to share their experience with colleagues together. Out of all teachers participating in teacher training during the pilot years, 60% have talked about the training experience with colleagues and 16% have carried out school-based training [41]. Teachers reported that the supportive and ready-to-use materials received from the training have been invaluable in implementing the PA activities in lessons [41].

##### Developing intervention tools for active recess

During the first pilot year, the focus of the seminars of active recess was on introducing possibilities for how to make the indoor and outdoor environment more PA friendly, open up gyms and sports halls during recess, and provide PA equipment (e.g. balls, racket games, etc.) for the students to use. As outdoor recess is not common in Estonia, schools were encouraged to try all-year outdoor recess as previous research had indicated the positive effect of outdoor recess on PA [42–45]. However, this raised the question of the outdoor infrastructure and affordances. Co-operation with architects was applied and supportive materials were developed [46]. Providing outdoor recess is a great challenge for the schools, and thus the change takes time – within three years two schools out of ten managed to implement a year-round outdoor recess.

The regulations concerning the outdoor recess are also crucial as, in Estonia, the weather conditions are cool and wet for most of the academic year and require appropriate clothing. This means that the students have to go to their wardrobe to get outdoor clothes, which is time-consuming and might require additional cleaning services as the students carry in dirt. Thus, the outdoor recess can bring additional costs that need to be acknowledged and pre-planned (see Tips 4 and 9 in Table 2). Some schools have made longer recess breaks in the middle of the school day and some school principals have made going out mandatory for the students. The latter intervention has turned out to be somewhat complicated because it may initiate complaints among the older students. In one inspiration seminar, a school principal talked honestly about the problems in implementing outdoor recess and how she solved them through negotiations with the students and re-designing the school rules by involving students. Now the students follow the school rules with greater enthusiasm because they have participated in the creation process (see Tip 9 in Table 2). This emphasized the necessity of the co-creation and supporting motivation [24] in multiple level – researchers with school principals, and teachers and school representatives with students, which constitutes prerequisite for unity and entirety for whole school approach.

During the first pilot year it became increasingly evident that students are an unused resource in the school, which could reduce the workload of teachers in organizing recess activities. Therefore, the focus of active recess training shifted to developing and implementing a training session for play leaders, who would organize PA activities and games during recess for younger students. At first play leaders’ training consisted of two consecutive days where students were taught the principles of organizing and carrying out a game, how to plan activities, which games to choose, and how to invite others to play. All participants also received a personal book of physically active games and each school was provided a set of small equipment used for active recess. During the pilot phase more than 295 play leaders from 39 network schools were trained. Based on the feedback from students and schools and to make the training available to more schools, in 2019 the length of the training of play leaders was reduced to one day. The play leaders’ system has been well received by the schools and students. However, during implementation, play leaders need support, help and guidance from some school personnel and thus one support person should also attend the training with children.

##### Action research

A co-design and practice-to-evidence approach needs action research. This includes constant research on the functionality of program elements and efficient monitoring of the occurring changes. The development process of SiM can also be described as flexible given that, after the development of a new material, method and/or seminar/training session by researchers and/or education experts, these elements were piloted and invaluable feedback concerning the applicability, necessity, sustainability and importance was received. Based on the needs of the schools, the developed elements have been either improved and incorporated to the model or discarded, thus, adjusting the program has become a part of action research. For the RDT, the important elements of obtaining the feedback and input from schools are different monitoring tools, e.g. a web-based questionnaire and self-evaluation tool for schools, personal communication and action research (see Tips 6 and 10 in Table 2).

For monitoring overall changes in attitudes and possibilities for PA, a web-based questionnaire was applied for students aged 10–16 years and school personnel. Moreover, a self-evaluation tool filled by school SiM teams was developed. Both tools helped to monitor the activities and changes in schools, plan further developments and at the same time served as an input to SiM teams in planning school action plan as all schools received a school-based feedback.

We have constantly collected qualitative data and kept in close contact with schools through visits and seminars. In addition to gaining insights, this closer contact with schools and (qualitative) research material consisting of examples or stories acts as a strong motivating factor for the RDT. Although this might only seem to be a positive side-effect, this could be a key driver for the RDT as the efficient intervention planning undoubtedly requires a great deal of engagement. Having occasional reminders in the form of comments, stories, and personal experience from school visits and observations is a highly needed impetus for this type of program. The collected qualitative data complements and helps to interpret quantitative data as the complexity of social situations and the multiplicity of factors makes it very difficult to ascertain causal relationships with only objective physical activity measurements or surveys (see Tip 6 in Table 2).

Main methods and findings from development and pilot phase of SiM program are described in Table 1.

**Table 1:** Main methods and findings from development and pilot phase of Schools in Motion (SiM) program

Time	Method	Main finding/ input to program
2014–2016	Literature review Team brainstorming	Schools are promising venues for intervention as children with different socio-economic background can be reached. The period between 6 and 12 years is critical in forming PA habits. Need for multi-component change program towards PA-friendly everyday arrangement of school life, practices of school staff and students. Sustainability of changes as main aim.
2015	Focus group studies	Schools are interested in encouraging students PA during the school day in addition to PE classes. To encourage PA, the modification of rules, re-design of space, tools and training for development of new practices are needed in schools.
2015	Objective PA measurements	Overall PA levels are low and the potential of school setting is underused
since 2016	Action research (incl. Conversations with school staff members and students; observations; analysis of Facebook group posts and action plans of schools; analysis of the feedback from inspirational seminars arranged for the schools within SiM program)	Each school in unique and therefore, the solutions and focuses of the schools are different. Supporting the autonomy of schools is a key element of reducing the odds of dropout. The changes in school are possible when there is a team leading the SiM activities and management is included.
2016–2019	Piloting of materials and tools for active lessons, active recess and SiM teams: analysis of the feedback from training seminars and participants’ diaries	Providing ready-to-use materials increase the implementation of new methods and SiM principles
2017–2019	Piloting play-leaders training sub-program: analysis of feedback from training camps and interviews with participants	Involvement of students and development of their training and rotation increases sustainability of intervention program
Since 2017	Self-evaluation tool	Changes in school culture take time but they are possible. Providing fast and simple feedback to schools about their results is a great motivation for them to continue.
2016–2019	Web-based questionnaire	The answers of school staff and younger students indicate the gradual, but not linear change towards PA-friendly school culture. In addition to providing fast and simple feedback to schools, it also provides important input to our program development. Selection and adaptation of the most promising indicators for PA-friendly school culture which was included in the 2020 national school satisfaction survey.

##### Communicative support and advocacy

In providing the necessary public support and awareness of the problem and potential solution in the form of SiM, public communication as well as direct advocacy have been very important. In SiM, we have largely framed the low level of PA as an urgent health problem. For example, to attract the attention of funders and the broader public, we presented children’s inactivity as the epidemic disease for the twenty-first century – it is a common message in newspaper articles and presentations. This framing has become an important rationale for the program. The medical facts and studies about positive associations between PA and mental (with a focus to learning achievements) and social health are constantly presented. In addition, the necessity for a more active class environment which is based on the arguments related to health effects and supported by medical experiments that observed the “acute effects of a simulated school day with reduced sitting or usual sitting on adolescents’ cognitive function and cardiometabolic biomarkers” [47] is highlighted. Via public communication of the SiM program, we questioned the existing normative beliefs that justify the status quo of long consecutive sitting in school settings, e.g. quiet sitting is good for academic achievement, PA means good performance in sports, schools who are oriented to high academic performance do not deal with activity breaks during lessons, etc. In so doing, we tried to avoid the purely medicalized framing [48] and represent the logic for intervention as social, psychological and cultural (see Tips 2 and 3 in Table 2). Additionally, the international Physical Activity Report Card [49] was developed in 2016 [50] and 2018 [51], accompanying a lot of media attention.

**Table 2:** The main tips and lessons learnt from the Estonian Schools in Motion (SiM) program design

No	Tip/lesson	Explanation	Example
**1**	**Compose multi-disciplinary RDT team and involve practitioners**	Involve into the team practitioners who have lengthy experience in working with or in schools and are familiar with the day-to-day operation of schools. They have certain tacit knowledge to anticipate whether the new intervention idea could fit with the implicit rules and logic of action in the (particular) school.	The team has agreed upon the main approach formed as a result of the site visits, close contact with schools and critical mapping of the earlier interventions, according to which it is not wise to set strict norms to schools in the way they are set in trial interventions. Instead, a flexible approach based on the principles of co-design of program elements with schools and supporting the autonomy of schools was approved.
**2**	**Involve the experts of public communication into the team**	The media representation and general recognition of the problem is influential in shaping the understandings of all key stakeholders, including parents, teachers and the local municipality. In order to include the topic in the media agenda, systematic communication is needed that works best when the communication experts are part of the team, rather than involved as an outsourced service.	Through public communication the concept of a PA-friendly school has been gradually normalized in society. Some schools have made successful fundraising via participatory budget projects; sports-, health- and educational organizations embraced the SiM approach and are interested in co-operation.
**3**	**Make multiple positioning of problems and solutions**	In analyzing the problem and discussing the solutions be aware of several standpoints and potential framings: health, pedagogy, schools traditions, social relationships, wellbeing, sport, etc. Do not let one meaning/positioning dominate and stifle other meanings/positionings. Include school personnel as a target group for PA promotion.	The health application alone does not give input into the necessity of PA during the school day. The pedagogical implications of the activity break in lessons and active recess are important to address and multiple solutions need to be encouraged. In the beginning of the program, the schools generally believed that academic results can best be achieved by sitting calmly and PA is mainly for physical education and after-school time. The communication based on scientific evidence assured them about positive supportive relationships between PA and academic advancement.
**4**	**Encourage implementers to focus on the long-term mobilization of resources**	The short-term mobilization (such as a sports day) is achievable and implementers like to fill their plans with one-off activities. Encourage patiently the implementers to compose their action plans more from the regular activities and changes, thus creating long-term impact. Encourage them to be rather conservative: plan fewer activities, but cover them with sufficient resources (people, time, regulations, etc.) for implementation. The measurement of the impact of the intervention has to involve both a short-term and long-term perspective.	The re-structuring of the school day or school physical environment is a substantive change that has several co-effects. In some schools it took 3–4 years before they made this change. From both implementers’ and program leaders’ viewpoint, planning would need more than a 1–2 year perspective as sustainable changes take time.
**5**	**Nurture openness and learning from negative experiences**	The schools are eager for positive self-representation and cautious of talking openly about their failures. It is possible to create an inspiration community both online (e.g. a Facebook group) and offline (e.g. experience-sharing seminars). This requires special efforts to transform the inspiration community into a learning community where failures are also discussed openly. The initiators need support and positive feedback.	In one inspiration seminar, a school principal talked openly about problems in implementing outdoor recess and how she solved the problem by involving students into the re-design of the house rules. This experience also give inspiration to the other schools and the research team for further improvements of the program.
**6**	**Close contact, qualitative data help to monitor and set the course for the program**	In the course of implementation new challenges appear constantly. In order to understand the implicit mechanisms, a deeper look is needed. The examples and stories have many functions.	Although we were hesitant about school visits at the beginning of the program, they have proven to have high functionality in diverse domains: to understand school culture; monitor the general progress of the school; and support the team and program with examples and stories.
**7**	**Plan time for practicing between seminars and workshops and request the participants to record their experience**	Teachers often feel inspired by new ideas and activities they have learnt in seminars or workshops and want to try them in their classrooms. However, the initial enthusiasm often tends to fade. Requests to record their experiences (keep a diary, take photos, etc.) over a certain period exert slight external pressure to keep on practicing and follow-up meetings boost motivation. Afterwards, the ideas are a fruitful base for designing the web-based database.	The teachers participating in the skills training were requested to keep a diary of the movement integration in their classrooms. On the second training day (follow-up meeting) they could exchange ideas and experience and were introduced to many new activities promoted by themselves.
**8**	**Give some instantly usable tools to aid practicing the newly acquired skills**	Teachers often feel that the preparation of physically active lessons is time-consuming. While it may largely be a misconception and many activities do not need many materials or special preparation, the ready-made and instantly usable materials increase the likelihood that the teachers who do not feel very experienced in using physically active methods try them out.	The teachers participating in the active lesson training received some ready-made materials on both training days. They could instantly use these materials in their classroom on the next day at school for implementing activity breaks or to integrate PA into learning.
**9**	**Involve different staff members and students into the planning meetings and program implementation and support their rotation**	Remind the activists that they should involve representatives of varied roles – from the principal to the cloakroom employee and students from different age groups – in planning the new interventions. The details on implementation should already be discussed when planning the recourse demanding changes. In the planning process it is really important to involve the students. Otherwise, the revenues from investment remain modest. Additionally, there is a danger of their burnout and limitations of their power to create and support changes. Motivate the new members to join the activist group by offering socialization tools/events to the newcomers.	The outdoor recess needs the establishment of a longer time break and a solution to problems of access to the wardrobe, additional cleaning, and new activity spaces in the schoolyard, and therefore needs careful planning involving different staff members of schools. For students it has been important that they are partners in developing the process in order to perceive outdoor recess as not just as the teachers’ order, but to co-design with them to create more physically active and enjoyable recess time. The motivation of students to be leaders of recess can be an important cue for older students.
**10**	**After conducting a study give instant and easy-to-use individual feedback to schools**	Conducting research creates an extra workload for the schools, and the least that can be done is appealing and easy-to-understand individual feedback which schools can use for analysis and monitoring. This approach ensures that the results will actually be used by the target group and encourages co-operation in the future. For some schools, comparative graphs can be triggers for making changes. However, comparative feedback must be presented in a sensitive way, e.g. using codes for school names where each school knows only its code.	Within one month after participating in research, all participating schools received individual feedback. In the case of individual PA measurement, all participants received individual feedback and schools received aggregated feedback. The feedback generally consisted of graphs. The school feedback was in the form of a slideshow that also served as a communication tool for the principal when introducing the results to the whole school. As a result of the personalized, individual and attractive feedback, the schools have been very eager to participate in research and are even asking for more research.

In 2018, a SiM webpage (www.liikumakutsuvkool.ee) was launched to communicate SiM activities and principles to a broader audience, such as schools outside the network, different stakeholders and interest groups. The webpage includes an overview of all SiM elements, tools and materials, coming events and a “Bank of Ideas” which is a database of methods, ideas and games on how to add PA to lessons, recess and environment.

##### Partnership with ministries and other partners

There are also numerous interest groups and stakeholders that are strongly related to the SiM’s aims and activities. To initiate major changes in schools and ensure that the changes are sustainable, schools need support from municipalities and parents. Thus, it is necessary that the general norms in the society support the activities of the schools, that parents and local governments are aware of multiple benefits to health and wellbeing that physically active school day can evoke, and that the stakeholders are supportive of the program. In parallel with SiM program development, the RDT has been searching for and initiating co-operation with different interest groups, e.g. ministries, municipalities, and governmental institutions responsible for public health and traffic safety, architects, sport associations, higher education institutions, etc. Co-operation has been established with some municipalities that have provided additional support for local schools and initiated the process of joining the network. On a state level our main partner throughout the development process has been the Ministry of Social Affairs and strong support towards our activities has been expressed by the Ministry of Education and Science and the Ministry of Culture.

RDT has devoted significant resources to communication for successful results in advocacy and in creating a meaningful partnership with authorities and organizations. This was achieved by creating and using the means of strategic communication, focusing on message creation and societal dialogue, designing and implementing events and other communication plans, finding outputs in the media and social media, and using a wide variety of other communication tools.

#### Lessons learnt and primary indicators of success

The primary indicators of success of SiM program for RDT are the willingness of schools to participate in co-creation of program development, the program popularity among schools and no dropout schools, strong partnership with ministries which enables to actively participate in policy making. All these tendencies help to pave the school culture which supports the physical activity of children.

Deriving from diverse phases and activities in the design process, we can offer some tips and suggestions that have been evaluated and tested during the SiM program. As the local contexts for a school-related PA-program are diverse, we have principally selected tactical suggestions that are not highly context-dependent, such as the nuances of education systems or existing PA practices among children.

The evaluation of the SiM program can be currently done mainly in terms of outputs not in terms outcomes nor long term social impact. The reflection of the program design process provided information about how to create the intervening materials, environments etc. in close co-operation with participants themselves, considering the complexity of their everyday life – the natural site where the PA-initiatives have to embed. Authors argue that this question is under-discussion so far among scholars and practitioners [52].

The SiM program utilized main principles of action research, i.e. each school got own performance report containing the results of web survey among school staff and students. These reports were provided to all participating schools very quickly. All schools also got a feedback to their action plans. Thus, the interaction and exchange of the feedback was mutual and iterative. There are some primary outcomes indicating the success of this participatory approach – no school have dropped out of the program, schools have completed action plans, school principals have expressed their interest to make systemic pro-PA changes in their schools. Also, SiM has strong co-operation with relevant ministries who ask contribution to the improvements to the regulations and educational, health and sport policies strategies regarding the school life. Numerous schools express their interest to join to the SiM program although this program does not offer any material incentives. In conclusion, we can consider the SiM program as vital.

A full discussion of program effectiveness lies beyond the scope of this article as the main aim of current article is to describe the design process. All measurements and studies conducted have given input to the development process, and have not yet been published. However, there is a plan to fill this gap in near future.

#### Future challenges

From 2019 the program has moved to the next step of scaling up the program for the newcomers and maintaining the changes SiM has initiated in the schools who have entered to the program in earlier years. The sustainability of the intervention is crucial for the success. The program should address the spillover effects that threaten to diminish the PA-related changes despite the good will of (leading) implementers. Consequently, not only the effectiveness promised by the trials but also the prospective vitality of the suggested intervention in the “natural” settings have to be addressed in the design of the program, as Reis et al. [10] have stressed in their seminal article. An important condition for the sustainability is the wide partnership, even power coalition [11] between schools, local governments, ministries, professional institutions, city planners, architects, sport institutions, universities who see the physically active school culture as new normality and act – with everybody within own functional roles – according to this normality.

Despite the positive acceptance of SiM by schools, there are still many challenges ahead. The RDT has analyzed shortages in the program and continues to work out solutions. The further development has to be more age specific. Outdoor recess and playing during recess are attractive for younger children but not appealing for the older students aged 13–16, who are the most physically inactive group in basic schools. The training of new skills among teachers has to involve not only the development of personal skills, but also knowledge transfer to the other teachers and staff members at the in-school training sessions, inspiration seminars, regional training networks or teacher training institutions. The PA-friendly school concept also considers teachers’ and principals’ health and PA, and students’ families’ PA improvement. The social norms that discourage active transport from home to school as a sign of parental negligence are also a great challenge to meet via public communication during the coming years. The further development of SiM and its scaling-up will proceed in parallel, accompanied by action research. In addition, the rapid growth of participants requires creating a sustainable model for managing the network, providing enough support for participating schools and, concurrently, to keep the main principle for co-creative and collaborative approach throughout scaling-up process.

## Conclusions

The development and implementation of a comprehensive school-based PA intervention is a great challenge as the school is a complex social network comprising different interests, stakeholders and aims. We have managed to develop a flexible and comprehensive school-based PA program which has been well received by both schools and overall society. However, many challenges still lie ahead. We believe that sharing our experience – both our successes as well as the difficulties faced, and lessons learnt – will help to promote the design and implementation PA-enhancing interventions within school system and fight the overwhelming physical inactivity epidemic worldwide.

## Data Availability

Data sharing is not applicable to this article as no datasets were generated or analyzed for the current study.

## Abbreviations

SiM: Schools in Motion
PA: Physical activity;
WHO: World Health Organization
MVPA: Moderate-to-vigorous physical activity
RDT: Research and development team
SEM: Socio-ecological model
SDT: Self-determination theory
IM: Intervention Mapping
